# Elevated Type 2 Inflammatory Factors, Th2/Th1 Balanced Status, and Exosomes as a Marker of Severity in Chronic Actinic Dermatitis

**DOI:** 10.1155/mi/7967853

**Published:** 2025-01-31

**Authors:** Tang Jun-Ting, Tu Ying, Nong Xiang, Sun Dong-Jie, He Li

**Affiliations:** Department of Dermatology and Venerology, The First Affiliated Hospital of Kunming Medical University, Kunming 650000, China

**Keywords:** atopic dermatitis, chronic actinic dermatitis, exosomes, inflammatory mechanisms, Th2/Th1

## Abstract

**Background:** Chronic actinic dermatitis (CAD) is a skin inflammation triggered by light exposure, occurring at the exposed site and potentially causing widespread inflammation throughout the body. While we hypothesize that severe CAD could progress to atopic dermatitis, the exact inflammatory mechanisms and pathogenesis remain unclear.

**Objective:** We aimed to investigate the relationships between CAD severity and clinical and immunological parameters.

**Methods:** CAD patients were classified into two groups based on severity: mild CAD and severe CAD. We assessed total IgE levels, eosinophil count in peripheral blood (PB), the ratio of Th2 cell percentage to Th1 cell percentages (Th2/Th1), and cytokine/chemokine levels in both PB and skin lesions.

**Results:** In our study, eosinophil counts in patients with severe CAD and mild CAD were significantly higher than those in the control group (*p*  < 0.05). It was exhibited a higher Th2/Th1 ratio in severe-CAD patients compared with mild-CAD patients and control group (*p*  < 0.05). There were significant increases in the levels of IL-4, IL-5, IL-8, IL-31, and IFN-*γ* in the lesions of severe CAD patients compared to the control group (*p*  < 0.05). Additionally, the level of CD63 exosomes in the PB of severe-CAD patients was significantly elevated compared to the control group (*p*  < 0.05). Persistent elevations of CD63 exosomes in severe CAD patients were associated with the Th2/Th1 balance status in PB (*p*  < 0.05).

**Conclusion:** Severe CAD demonstrates a shift toward Th2 immunity from Th2/Th1, accompanied by elevated with inflammatory factors such as IL-4, IL-5, IL-31, IL-8, and IFN-*γ* in skin lesions, as well as increased CD63 exosomes in PB. Thus, consequently, exosomes and Th2/Th1 imbalance may contribute to the systemic manifestations observed in CAD patients.

## 1. Introduction

Chronic actinic dermatitis (CAD) is a rare photosensitive disorder that predominantly affects elderly men. CAD patients commonly live in high-altitude areas [1]. It demonstrates heightened sensitivity to a broad spectrum of light, including ultraviolet B (UVB), ultraviolet A (UVA), and visible light [2]. Typically, CAD manifests on sun-exposed areas such as the face, neck, scalp, and limbs, presenting with “lion face” facial skin changes. During flare-ups, the lesions can extend to the torso, resembling atopic dermatitis (AD). CAD patient usually requires the administration of oral steroids or immunosuppressants such as azathioprine. However, most patients are prone to recurrent with low treatment effect. Notably, the use of Dupilumab, a monoclonal antibody targeting interleukin-4 (IL-4) and interleukin-13 (IL-13) [3], has shown promising therapeutic outcomes for severe and widespread CAD cases. Therefore, we believe that biological agents such as IL-4R inhibitors have good effects in the treatment of CAD through regulating Th2 cells and Th2 cell-related cytokines. Previous research highlights the involvement of major inflammatory mediators such as IL-8 and the type 1 immunity marker IFN-*γ* in CAD pathogenesis [4–6]. Clinical observations suggest that type 2 inflammatory cytokines also play a role in CAD, mirroring the mechanisms observed in AD. AD is a chronic inflammatory skin condition characterized by itching, redness, and compromised skin barrier function [7], Alarmins including IL-25, IL-33, and thymic stromal lymphopoietin (TSLP) activate type 2 innate lymphoid cells, which release cytokines like IL-4, IL-5, and IL-13, crucial in the pathogenesis of AD [8]. Due to severe CAD and AD shared clinical features, severe CAD has often been likened to AD. This observation prompted us to hypothesize that CAD may involve Th2 cells and could progress to AD.

Recently, exosomes have gained attention as crucial mediators of intercellular communication, influencing inflammation, angiogenesis, fibrosis, and tissue regeneration [9]. Exosomes expressing tetraspanins such as CD63, CD9, and CD81 serve as distinctive biomarkers with high specificity [10]. Cho BS found that subcutaneous injection of adipose tissue-derived stem cell exosomes in an AD model remarkably reduced the levels of inflammatory cytokines such as IL-4, IL-5, IL-13, TNF-*α*, IFN-*γ*, IL-17, and TSLP. This finding suggests a close relationship between exosomes and type 2 inflammatory cytokines in severe CAD [11]. Therefore, we will identify CAD by detecting CAD-related cytokines/chemokines and exosomes assess the inflammatory pattern and determine whether there are conditions such as Th2/Th1 immune drift, and find new cytokines/chemokines and exosomes that have the director to better evaluate mild CAD and severe CAD, seeking better therapeutic target drugs.

## 2. Materials and Methods

### 2.1. Clinical Information

We recruited individuals diagnosed with CAD and an equal number of healthy subjects matched for sex and age. Clinical blood analyses were conducted during each visit involving 19 male participants. The average age of healthy subjects was 50.5 years (range 46.5−56.5). CAD severity was categorized into mild CAD and severe CAD based on the affected sites and the Eczema Area and Severity Index (EASI) score. Mild CAD was 58 years (range 52−69), and severe CAD was 61.5 years (range 45.7−76.7). Blood samples of 100 mL per subject were drawn from the antecubital vein of both CAD patients and healthy subjects, and skin samples were collected from both groups. This study was approved by the Ethics Committee of the First Affiliated Hospital of Kunming Medical University (Approval No. 2022L-20). All participants signed the informed consent. Clinical information was abstracted from medical records. Data of Eosinophils (Eos) and IgE levels were collected from medical records.

### 2.2. Preparation of Skin Single Cell Suspension

Skin tissues obtained from both CAD patients and healthy subjects were rinsed in a 1 × PBS solution precooled to 4°C to remove unwanted tissue components were eliminated. The skin was then cut into particles of 0.5 cm^3^ in size and washed to remove debris. The treated skin tissue was placed into a centrifuge tube containing 15 mL of RPMI 1640 (Gibco) with 2 U/mL Dispase II (Sigma) [12]. This tube, with the enzymatic solution and skin tissue, was immersed in a water bath set at 37°C and rotated at 60 rpm/min for digestion, maintaining this condition for 1 h. The epidermis was transferred to a cell culture dish containing 15 mL of RF10 (RPMI + 10% FBS + 1% Penicillin–Streptomycin + 1% L-Glutamine). Next, 1.6 mg/mL collagenase II (Solarbio) was added, and the dish was incubated in a 37°C, 5% CO_2_ incubator for overnight, or more than 12 h. Subsequently, the supernatant was collected using a pipette and filtered through a 100 μm pore size filter.

### 2.3. Preparation of PBMCs From Venous Blood

Peripheral venous blood was collected into K2EDTA tube. The PBMCs were isolated using density gradient centrifugation with Lympholyte H Cell Separation Medium (TBD). The remaining platelets and erythrocytes were removed using red blood cell lysis (solarbio) 350 g centrifuged for 10 min, respectively. The supernatant was abandoned, and the culture medium was completely suspended. The isolated PBMCs were stored in liquid nitrogen at a density of 1–10 × 10^6^ cells/mL.

### 2.4. Th2/Th1 Cell Detection in Venous Blood and Skin

To assess lymphocyte response, these cells from skin tissues or peripheral venous blood were stimulated in a 96-well plate, coated sequentially with a cell-stimulating mixture, protein transport inhibitor (2 μg/mL; eBioscience), for 6 h at 37°C. Following stimulation, the cells were stained using the Human TruStain FcXTM kit (5 μL; Biolegend), followed by anti-CD4-PE-cy7 (5 μL/test; Biolegend) and anti-IFN-*γ*-FITC (5 μL/test; Biolegend) for Th1 cell analysis. Additionally, anti-CD4-PE-Cy7 (5 μL/test; Biolegend) and anti-IL-4-PE (1.25 μL/test; Biolegend) were used for Th2 cell analysis. Subsequently, FACS analyses were performed.

### 2.5. Cytokine/Chemokine Analyses in Venous Blood and Skin

Samples of venous blood and skin were collected from both CAD patients and healthy subjects. To assess cytokines/chemokines from skin, tissue specimens were divided into small pieces and mechanically disrupted in the presence of an isolation medium. Isolated from the cell suspension by density centrifugation on ice bath with 300 μL of PBS medium (Wisent) at 4°C 12,500 g, for 10 min. To assess cytokines/chemokines from venous blood, serum was isolated from peripheral blood (PB). Cytokines/chemokines including IL-4, IL-5, IL-13, IFN-*γ*, IL-8, IL-25, IL-31, IL-33, TSLP, and CD63 were analyzed using ELISA.

## 3. Results

### 3.1. CAD Evaluation and Classification by EASI Score

**Figure 1:**
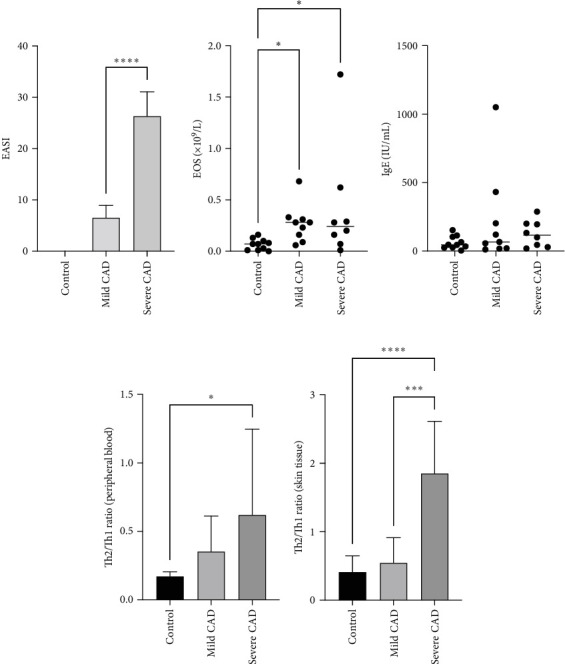
Assessment of Th2/Th1 shift, Eos, and IgE levels among three groups. The patients were divided into severe CAD, mild CAD, and control group by EASI Score evaluation, and the Th2/Th1 ratio in peripheral blood or skin was evaluated by flow cytometry, Eos and IgE levels were collected from clinical data. (A) Through EASI Score evaluation, divided to severe CAD, mild CAD, and control group, EASI Score in severe CAD was significantly higher than that of mild CAD. (B) Eos levels in mild CAD and severe CAD were higher than that of control group. (C) There was no significantly difference in IgE levels among three groups. (D) Th2/Th1 ratio of peripheral blood was significantly increased in severe CAD when compared to control group. (E) Th2/Th1 ratio of skin was significantly increased in severe CAD when compared to control group and mild CAD. Statistical analyses were performed using one-way ANOVA and LSD test or Kruskal–Wallis test. *⁣*^*∗*^*p*  < 0.05, *⁣*^*∗∗*^*p*  < 0.01, *⁣*^*∗∗∗*^*p*  < 0.001, and *⁣*^*∗∗∗∗*^*p* < 0.0001.

Through EASI Score evaluation divided to mild CAD (6.53 ± 2.40) and severe CAD (26.35 ± 4.73), EASI Score of severe CAD was significantly higher than that of mild CAD (*p*  < 0.0001) (Figure 1A).

### 3.2. The Eos and IgE Levels in PB of CAD Patients

Eos and IgE levels in PB of patients were collected from clinical data. The results indicated a significant increase in Eos in severe-CAD patients 0.24 (0.09,0.53) and mild-CAD patients 0.28 (0.13, 0.32) compared to the control group 0.07 (0.01, 0.11) (*p*  < 0.039, *p*  < 0.014). No significant difference was observed between patients in the mild group and severe group (*p*  > 0.05) (Figure 1B). However, there was no difference among the three groups in IgE levels (*p*  > 0.05) (Figure 1C).

### 3.3. Th2/Th1 Ratio in PBMC and Skin Lesions of CAD Patients

The ratio of Th2 cell (IL4^+^ CD4^+^) percentage to Th1 cell (IFN-*γ*^+^ CD4^+^) percentage (Th2/Th1 ratio) in PB of CAD patients was compared based on CAD severity. In the severe group, all patients exhibited a higher Th2/Th1 ratio 0.41 (0.22, 0.91) compared to the Th2/Th1 balanced status in the normal control group in PB 0.17 (0.16, 0.19) (*p*=0.02). No significant difference was observed between patients in the moderate group 0.28 (0.20, 0.42) and the control group (*p*=0.07). No significant difference was observed between patients in the moderate group 0.28 (0.20, 0.42) and the severe group (*p*  > 0.05) (Figure 1D).

The ratio of Th2 cell (IL4^+^ CD4^+^) percentage to Th1 cell (IFN-*γ*^+^ CD4^+^) percentage (Th2/Th1 ratio) in CAD patients' lesions was also compared based on CAD severity. In the severe group, all patients showed a higher Th2/Th1 ratio (1.85 ± 0.76) compared to the Th2/Th1 balanced status in the moderate group (0.54 ± 0.36) and normal control group (0.41 ± 0.23) (*p*  < 0.0001, *p*=0.0008) (Figure 1E). There was no significant difference between moderate group and control group (*p*  > 0.05).

### 3.4. Cytokine/Chemkine Levels in Skin Lesions of CAD Patients

**Figure 2:**
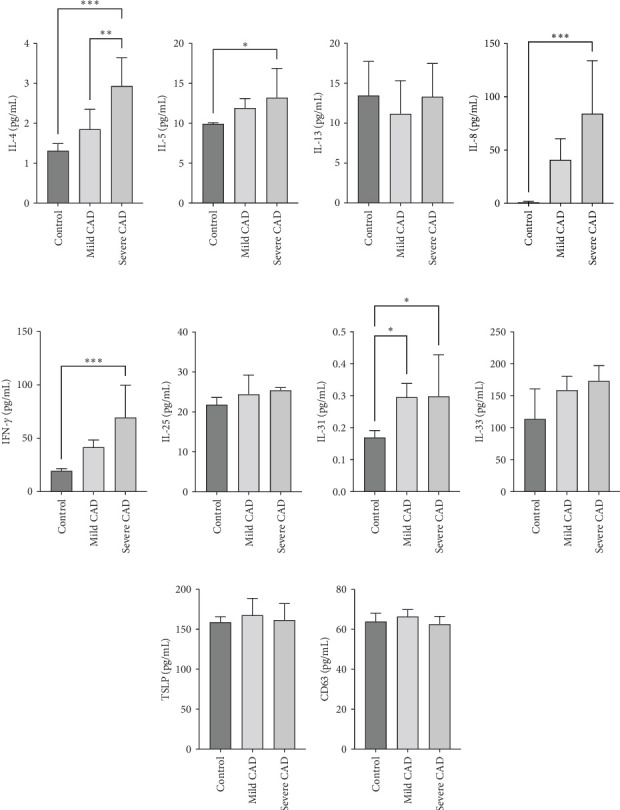
Cytokine/chemokine levels in Skin Lesions of mild CAD, severe CAD, and control group. IL-4 (A) significantly increased in severe CAD when compared to mild CAD and control group. IL-5 (B) IL-8 (D), IFN-*γ* (E), and IL-31 (G) significantly increased in severe-CAD patients when compared to control group. There were no significant differences in IL-13 (C), IL-25 (F), IL-33 (H), TSLP (I), and CD63 (J). Statistical analyses were performed using one-way ANOVA and LSD test or Kruskal–Wallis test. *⁣*^*∗*^*p*  < 0.05, *⁣*^*∗∗*^*p*  < 0.01, and *⁣*^*∗∗∗*^*p*  < 0.001.

Cytokine/Chemkine levels in skin lesions of CAD patients, including IL-4, IL-5, IL-13, IFN-*γ*, IL-8, IL-25, IL-31, IL-33, TSLP, and CD63, were measured using ELISA. The results revealed significantly higher levels of IL-4 (2.938 ± 0.71), IL-5 (13.21 ± 3.65), IL-31 (0.29 ± 0.13), IL-8 (84.24 ± 49.64), and IFN-*γ* (69.58 ± 30.28) in severe CAD patients' lesions compared to the control group (1.31 ± 0.17), (9.92 ± 0.16), (0.16 ± 0.02), (0.86 ± 0.82), and (19.52 ± 2.02) (*p*=0.0001, *p*=0.03, *p*=0.022, *p*=0.0004, *p*=0.0003). IL-4 levels were significantly higher in severe CAD patients' lesions than in mild CAD patients' lesions (0.29 ± 0.04) (*p*=0.009). IL-31 levels were significantly higher in mild CAD patients' lesions than in the control group (0.16 ± 0.02) (*p*=0.024). There were no significant difference among three groups in IL-25, IL-33, TSLP, and CD63 (*p*  > 0.05) (Figure 2A–J).

### 3.5. Serum Cytokine/Chemokine Levels in CAD Patients

**Figure 3:**
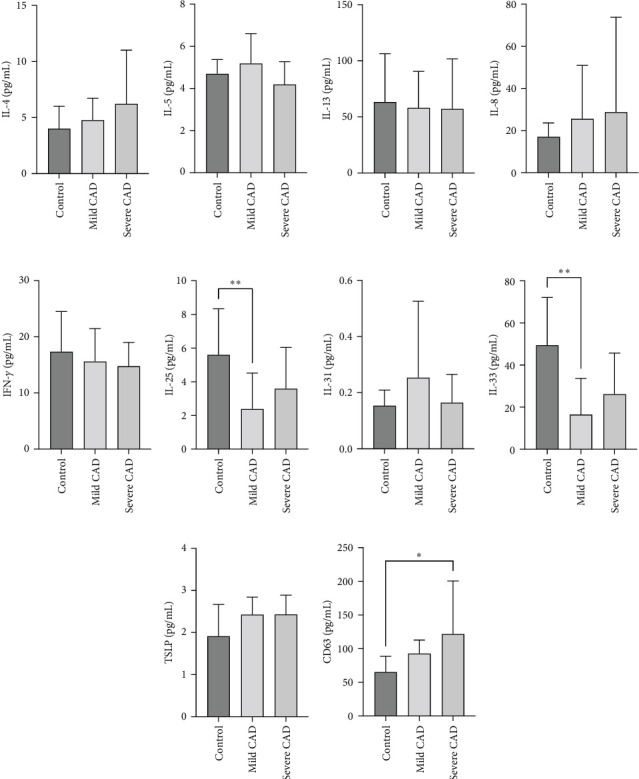
Serum cytokine/chemokine levels for CAD patients. CD63 (J) significantly increased in severe CAD when compared to mild CAD and control group. IL-25 (F) and IL-33 (H) significantly decreased in mild CAD when compared to control group. There were no significant differences in IL-4 (A), IL-5 (B), IL-13 (C), IL-8 (D), IFN-*γ* (E), IL-31 (G), and TSLP (I). Statistical analyses were performed using one-way ANOVA and LSD test or Kruskal–Wallis test. *⁣*^*∗*^*p*  < 0.05, *⁣*^*∗∗*^*p*  < 0.01, and *⁣*^*∗∗∗*^*p*  < 0.001.

Serum levels of IL-4, IL-5, IL-13, IFN-*γ*, IL-8, IL-25, IL-31, IL-33, TSLP, and CD63 were assessed using ELISA. The results indicated a significant increase in CD63 levels in severe-CAD patients (128.0 ± 87.95) compared to the control group (65.33 ± 23.38,) (*p*=0.029). No significant difference was observed between patients in the mild CAD-group (93.07 ± 24.46) and severe-CAD group (65.33 ± 23.38) (*p*=0.35).There was a significantly decrease in IL-25 in mild-CAD patients (2.35 ± 2.13) when compared to control group (5.605 ± 2.73) (*p*=0.009). There was a significant decrease in IL-33 in mild-CAD patients 7.38 (0.20, 34.21) when compared to control group 44.04 (37.16, 55.86) (*p*=0.0024). There were no significant difference among three groups in IL-4, IL-5, IL-13, IFN-*γ*, IL-8, IL-31, and TSLP (*p*  > 0.05) (Figure 3A–J).

### 3.6. Relationship Between Th2/Th1 Ratio and CD63 Levels in PBMC of CAD Patients

**Figure 4:**
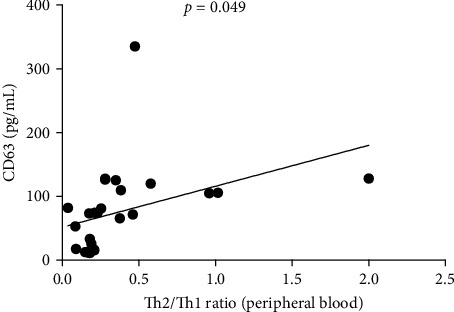
Correlation analysis of Th2/Th1 and exosomes. Th2/Th1 was significantly associated with exosomes (CD 63) in peripheral blood.

The ratio of Th2 cell (IL4^+^ CD4^+^) percentage to Th1 cell (IFN-*γ*^+^ CD4^+^) percentage (Th2/Th1 ratio) in CAD patients was related to CD63 (*p*  < 0.049) (Figure 4), there was no correlation between Th2/Th1 ratio and CD63 in lesions (data not shown).

### 3.7. Statistical Analysis

In the descriptive statistics, statistical analyses were performed using GraphPad Prism 9.0. Normally distributed data were presented as average and standard deviations (SDs). Data with skewness distribution were presented as medium and interquartile ranges. One-way ANOVA or Kruskal–Wallis test was used for analyses among the three groups. Only independent two groups were compared using the *t*-test or Mann–Whitney *U* test. Correlation analysis was using simple linear regression. *p*  < 0.05 was considered statistically significant.

## 4. Discussion

Our study revealed a notable shift toward Th2 immunity from the Th1/Th2 balanced status, particularly evident among severe-CAD patients, consistent with reported findings [13]. Recent evidence suggests that UV radiation induces the aggregation and chemotaxis of mast cells, dendritic cells, or neutrophils to skin tissue, triggering cytokine activation, including IL-25, IL-33, and TSLP activation, leading to T helper 2 polarization [14–16]. However, IL-25, IL-33, and TSLP levels were not found to be elevated in our CAD samples. Therefore, we propose that during the development of CAD, there is a shift of Th1 cells undergo a shift toward Th2 cells through mechanisms regulated by other cytokines.

We identified new cytokines involved in the mechanism of Th2 polarization in CAD patients. Cytokines IL-4 and IL-5, produced by Th2 cells, regulate IgE synthesis by secreting IL-4 to allergen-specific B cells and promote eosinophil-driven inflammation through IL-5 [17]. While IL-4 and IL-5 were present in the skin during the transition from mild CAD to severe CAD, and eosinophil levels were increased in blood, there were no significant differences in IgE levels among control, mild CAD, and severe CAD. Therefore, we believe that Th2 cell expansion may induce IL-4 and IL-5 release, contribute to eosinophil multiplication, leading to CAD aggravation. IL-31, a type 2 cytokine implicated in the itch sensation in AD [18], was elevated in both mild and severe CAD patients. Moderate upregulation of IL-31 mRNA expression was observed in epidermal keratinocytes and dermal fibroblasts following UVB exposure [19], indicating its role in the CAD mechanism. We also noted a significant increase IL-8 in severe CAD, a potent inflammatory chemokine IL-8 that recruits neutrophils to the skin [20]. Neubert E found long-wave ultraviolet light-induced Neutrophil extracellular trap formation [21]. Therefore, we speculated that IL-8 -mediated recruitment of neutrophils plays a critical role in the pathogenesis of severe CAD. IFN-*γ*, which stimulates Th1 cell proliferation, is involved in CAD development. Gamze P found that, as severe-CAD progresses, a shift from type 1 to type 2 cytokines is observed, characterized by less IFN-*γ* and more IL-4 expression following UVB irradiation in human skin experiments [22, 23]. Hasterok S indicates that ATG5 exacerbates UVB-induced keratinocyte ferroptosis in the epidermis, which subsequently gives rise to the secretion of IFN-*γ* [24]. Despite a relative decrease in Th1 cells in severe CAD, IFN-*γ* levels remained elevated, primarily due to activation of NKT cells and macrophages that release IFN-*γ* [25]. Thus, we propose that after following UVB radiation, increased expression of Th2 cells and cytokines such as IL-4, IL-5, IL-31, IL-8, and IFN-*γ* may contribute to lesion enlargement and severity of the lesion area in CAD patients. Recent studies underscore the pivotal role of Th2 cell-mediated type 2 immunity in AD, where inflammatory cytokines, like IL-4, IL-31, and IL-5, drive the pathogenesis of AD. Although the inflammatory profile in mild CAD differs from AD, a Th2/Th1 shift as lesions spread throughout the body suggests a pattern more akin to AD, potentially indicating the CAD progression toward AD development [26].

In our study, we investigated how the expression of Th2 cells contributes to the enlargement and spread of lesions in CAD. Our results revealed an increase in exosomes in the PB of severe CAD patients. Exosomes, a subtype of extracellular vesicles (EVs), are vesicles secreted by cells and can be found in various biological fluids, including blood. We utilized anti-CD63 antibodies conjugated to gold nanoparticles as an indicator for exosome detection [10]. Exosomes have been implicated in modulating Treg cells and activating T cells in inflammatory diseases such as ankylosing spondylitis (AS) and COVID-19 [27, 28]. They may serve as an indicator of disease severity. Although our study demonstrated a significant correlation between severe CAD and increased exosomal levels in PB of severe CAD, these levels did not correspond to CAD severity in affected tissues. It has been reported that the cargo within circulating exosomes can be transferred to skin cells via blood flow, potentially influencing the development of skin disorders [9]. This transfer might be linked to a surge in exosomal release into the bloodstream as rashes worsen from exposure to nonexposed areas. Additionally, while plasma exosomes were associated with the Th2/Th1 ratio, they showed no correlation with eosinophilia or total IgE levels. This suggests that exosome release may contribute to the enlargement of lesion areas in CAD patients, the question of whether exosomes induce Th2 polarization in the blood requires further in vitro verification. The increase in exosomes parallels the severity of CAD, consistent with observations in many allergic diseases. However, due to the limited sample size, it is essential to expand the sample size for further investigation in the future.

## 5. Conclusions

In conclusion, our findings highlight a correlation between severe CAD and a shift toward Th2 immunity from Th2/Th1, with exosomes and inflammatory factors, such as IL-4, IL-5, IL-31, IL-8, and IFN-*γ* in skin lesions, playing a crucial role in CAD development. These results underscore the potential of Th2 cells and exosomes as potential novel therapeutic targets for CAD, presenting a new concept for clinical diagnosis. CAD may be considered as a subtype of AD.

## Data Availability

Data used in this study are available upon request.

## Nomenclature

CAD: Chronic actinic dermatitis
PB: Peripheral blood
UVB: Ultraviolet B
UVA: Ultraviolet A
AD: Atopic dermatitis
IL-4: Interleukin-4
IL-13: Interleukin-13
IFN-*γ:*: Interferon-*γ*
EASI: Eczema Area and Severity Index
Eos: Eosinophils
TSLP: Thymic stromal lymphopoietin
EVs: Extracellular vesicles
AS: Ankylosing spondylitis.
